# Potential Diagnostic Error for Emergency Conditions, Mortality, and Healthy Days at Home

**DOI:** 10.1001/jamanetworkopen.2025.16400

**Published:** 2025-06-17

**Authors:** Michelle P. Lin, Ryan C. Burke, Amber K. Sabbatini, Ellen Latsko, Jonathan A. Edlow, E. John Orav, Laura G. Burke

**Affiliations:** 1 Department of Emergency Medicine, Stanford University, Palo Alto, California; 2 Department of Emergency Medicine, Beth Israel Deaconess Medical Center, Boston, Massachusetts; 3 Department of Emergency Medicine, Harvard Medical School, Boston, Massachusetts; 4 Department of Health Policy and Management, Harvard T.H. Chan School of Public Health, Boston, Massachusetts; 5 Department of Emergency Medicine, University of Washington, Seattle; 6 Division of General Internal Medicine, Brigham and Women’s Hospital, Boston, Massachusetts

## Abstract

**Importance:**

There is limited evidence regarding the frequency of diagnostic errors and outcomes associated with them in emergency care in the US.

**Objective:**

To examine rates of potential diagnostic errors and associated clinical outcomes among Medicare beneficiaries with emergency hospitalizations.

**Design, Setting, and Participants:**

This cohort study examined a national sample of fee-for-service Medicare beneficiaries aged 65 years or older with emergency hospitalizations for 10 high-risk conditions from 2016 to 2019. Data were analyzed from December 20, 2022, to April 16, 2025.

**Exposure:**

Potential diagnostic errors in the emergency department (ED).

**Main Outcome and Measures:**

The percentage of hospitalizations with an ED discharge (potential diagnostic error) in the preceding 9 days was calculated. From this observed ED visit rate, an adjusted rate of potential diagnostic errors was calculated by subtracting the rate of background ED discharges among a comparison cohort of beneficiaries with similar baseline risk. To examine the association between potential diagnostic error and outcomes, multivariable linear regression models were specified for the outcomes: 30-day mortality and healthy days at home (HDAH). Potential diagnostic error was the exposure, and the models were adjusted for diagnosis, patient demographics, and chronic conditions.

**Results:**

Among 302 837 emergency hospitalizations, 13 550 (4.5%) were preceded by an ED discharge (ie, potential diagnostic error) within 9 days. For the comparison group with similar baseline risk, 1.2% (95% CI, 1.2%-1.3%) had an ED discharge in a random 9-day period. After accounting for this background use, the adjusted potential diagnostic error rate was 3.2% (95% CI, 3.1%-3.3%) for all conditions in aggregate but ranged from 2.1% (95% CI, 1.9%-2.4%) for spontaneous intracranial hemorrhage to 15.6% (95% CI, 11.9%-19.3%) for spinal abscess. Having a potential diagnostic error was associated with higher adjusted 30-day mortality for emergency admissions (15.7% vs 14.9%; point absolute difference, 0.8 percentage points, 95% CI, 0.2 to 1.4 percentage points; *P* = .007) as well as fewer 30-day HDAH (13.5 days vs 15.0 days; difference, −1.4 days; 95% CI, −1.6 to −1.3 days; *P* < .001), although associations varied among conditions.

**Conclusions and Relevance:**

In this cohort study of Medicare beneficiaries aged 65 years and older with emergency hospitalizations, the adjusted potential diagnostic error rate was modest overall but varied by condition. Potential diagnostic errors were associated with worse outcomes for emergency conditions overall, with associations again varying among individual conditions.

## Key Points

**Question:**

How often are emergency hospitalizations preceded by an emergency department discharge among Medicare beneficiaries, and are these potential diagnostic errors associated with outcomes?

**Findings:**

In this cohort study of 302 837 emergency hospitalizations of fee-for-service Medicare beneficiaries, the adjusted estimate of potential diagnostic error within 9 days of an emergency hospitalization was 3.2%, with substantial variation by condition. Emergency hospitalizations with a potential diagnostic error had higher adjusted mortality and fewer healthy days at home at 30 days compared with admissions without a potential diagnostic error.

**Meaning:**

These findings suggest that while potential diagnostic errors were relatively infrequent for several high-risk emergency conditions, they were generally associated with worse outcomes.

## Teaser

This cohort study examines a national sample of Medicare beneficiaries aged 65 years or older with emergency hospitalizations for high-risk conditions from 2016 to 2019.

## Introduction

Diagnostic error, or inaccurate or delayed diagnosis that impacts management or adversely affects patient outcome,^1^ is a major public health problem. An estimated 12 million diagnostic errors occur in outpatient settings annually, with nearly half causing serious harm.^2^ The emergency department (ED) is thought to be a high-risk setting for diagnostic errors due to high patient volumes, clinician distractions, time-pressured decision-making, and uneven availability of resources (eg, specialty consultation). However, few studies have assessed how frequently potential diagnostic errors occur before emergency hospitalizations, or the extent to which they are associated with adverse outcomes.

A report by the Agency for Healthcare Research and Quality (AHRQ)^3^ suggested more than 7.4 million patients in EDs in the US may be misdiagnosed, with 2.6 million experiencing an adverse event. While the report has received significant media and policy attention, its overall estimates are extrapolated from 4 small studies^4,5,6,7^ conducted outside the US. Contemporary, empirical estimates of diagnostic error rates across a broad range of conditions in EDs in the US are currently limited. Large, administrative datasets have the potential to overcome some limitations of prior work on diagnostic errors,^8,9^ especially given that diagnostic errors may be more frequent for rare conditions. Medicare claims have numerous advantages for studying diagnostic error, including identification of return visits to a different institution and out-of-hospital deaths through linkage with the Social Security Death Index. However, no national studies to date have examined potential diagnostic error rates and associated outcomes across a broad range of high risk emergency conditions.

To address this evidence gap, we investigate the following questions. First, how often are Medicare beneficiaries hospitalized with 10 high-risk emergency conditions seen and discharged from the ED in the prior 9 days? Second, what proportion of these potential diagnostic errors preceding an emergency hospitalization reflect background, unrelated ED utilization rather than diagnostic errors? Third, to what degree are potential diagnostic errors in the ED associated with adverse outcomes, as measured by 30-day mortality and healthy days at home (HDAH)?

## Methods

This study was determined to be exempt, and informed consent was waived by the institutional review board at Beth Israel Deaconess Medical Center. This study follows the Strengthening the Reporting of Observational Studies in Epidemiology (STROBE) reporting guideline.

### Study Population and Hospitalizations

We examined a cohort of Medicare beneficiaries with emergent inpatient hospitalizations from 2016 to 2019 for 1 of the following high-risk, time-sensitive conditions: acute myocardial infarction, aortic aneurysm, aortic dissection, arterial thrombosis, ischemic stroke, meningitis or encephalitis, pulmonary embolism (PE), spinal abscess, spontaneous intracranial hemorrhage, and subarachnoid hemorrhage. These conditions were chosen because they were included in the AHRQ report on diagnostic error^3^ and have readily identifiable *International Statistical Classification of Diseases and Related Health Problems, Tenth Revision *(*ICD-10*) codes (eAppendix 1 in Supplement 1). Beneficiaries could have more than 1 inpatient stay in the sample, and each counted as a separate index admission.

This cohort was extracted from a random 20% sample of traditional Medicare beneficiaries aged 65 years and older in 2016 to 2019; we identified beneficiary age, sex, race (using the Research Triangle Institute Variable),^10^ Medicaid eligibility, and death date from the Master Beneficiary Summary File. Chronic conditions were obtained from the Chronic Conditions Warehouse (CCW) file. We identified beneficiary Centers for Medicare & Medicaid Services Hierarchical Condition Category (HCC) scores, which are associated with health care utilization and mortality.^11,12,13^ Both CCW conditions and HCC scores were identified from the prior calendar year to avoid adjusting for changes in health status that occurred during or after the index hospitalization. To account for the background rate of ED visits unrelated to diagnostic errors, we also examined ED visits among a comparison cohort of Medicare beneficiaries with similar clinical risk, as defined by HCC scores.

### Exposure

Our primary exposure was the presence of a potential diagnostic error, as defined by an ED discharge preceding an emergent hospitalization. We use the term potential diagnostic error because we did not have access to medical records to confirm the presence of an error.^14^ Additionally, some misdiagnoses are not preventable^15^ and may not be considered errors or violations of the standard of care by peers. Prior empirical research has suggested 9 days as the optimal time frame for capturing return ED visits associated with quality of care at the initial encounter.^16^ ED discharges were identified using facility and professional claims^17^ (eAppendix in Supplement 1). Prior ED visits could occur at any facility, not just the same hospital as the emergency admission. We excluded ED visits on the same or prior calendar day to avoid misclassifying interhospital transfers or duplicate records as potential diagnostic errors.

### Outcomes

We examined mortality within 30 days of admission. We chose 30 days because this time frame is commonly used to measure hospital quality^18,19,20^ and shorter periods may miss deaths following a prolonged hospitalization. We also examined healthy days at home (HDAH), an outcome measure that accounts for mortality as well as total time spent in acute and postacute care settings.^21^ Consistent with prior work,^22,23^ we subtracted from 30 the number of days from the index admission date during which the beneficiary was in 1 of the following settings: inpatient, observation, rehabilitation, ED, and long-term hospital. Additionally, if a beneficiary died during the 30-day period, the remaining days in the 30-day period after the death date were subtracted.

### Statistical Analysis

#### Estimating Rates of Potential Diagnostic Error

We calculated raw rates of potential diagnostic error with 9 days as the primary look-back period. Additionally, to generate appropriate 95% CIs that account for patient clustering within hospitals, we specified linear probability models with potential diagnostic errors as the outcome and incorporating hospital random effects.

#### Estimating Background ED Utilization Among the Comparison Cohort

For each Medicare beneficiary in the comparison cohort, we selected a random date, stratified by season. Thus, for each season, the proportion of dates for the comparison cohort and the proportion of emergency admissions for the diagnostic error cohort were the same. To allow for subsequent adjustment for complexity, the comparison cohort was divided into HCC subgroups with 0.05 increments, ranging from 0.80 to 1.60. Within each subgroup, we calculated the proportion of beneficiaries in the comparison cohort with an ED discharge in the 9 days preceding the random date. These rates represent estimates of the background 9-day ED utilization by patient complexity.

#### Estimating the Adjusted Incidence of Potential Diagnostic Errors

We estimated the adjusted incidence of potential diagnostic errors by subtracting the background 9-day ED visit rate from the 9-day potential diagnostic error rate in the emergency hospitalizations sample. We did this for emergency hospitalizations overall and by condition. The background rate was chosen from the HCC subgroup that contained the mean HCC score of the emergency hospitalization cohort.

#### Association Between Potential Diagnostic Errors and Outcomes

We specified a linear probability model for a beneficiary’s 30-day mortality as the outcome and presence of a potential diagnostic error as the binary estimator. We adjusted for hospital random effects, principal diagnosis, and year, as well as patient age, sex, Medicaid eligibility, and conditions from the CCW file. We specified analogous linear regression models for 30-day HDAH.

Data were analyzed from December 20, 2022, to April 16, 2025. Statistical significance was set at *P* < .05, and all tests were 2-sided. SAS version 9.4 (SAS for Statistical Computing) was used for analyses.

#### Sensitivity Analysis

While our 9-day look-back interval is supported by prior evidence,^16^ some studies have used other time periods (eg, 30 days).^24^ Thus, we calculated daily rates of potential diagnostic error, background ED utilization and adjusted potential diagnostic error out to 90 days and report these values overall and by condition at 14 and 30 days. Our primary approach excluded visits on the same or prior day as the hospitalization, as these likely represent transfers or duplicate records but we may also have missed some true ED returns within 24 hours. Thus, we repeated our calculations of potential diagnostic error using facility claims (eAppendix in Supplement 1), excluding only prior visits with a disposition status of transfer (regardless of time frame). Additionally, we repeated our models for the outcomes of 30-day mortality and HDAH using hospital fixed effects (vs random effects in the primary analyses), to examine the association between prior ED visits and outcomes among patients within the same hospital.

## Results

### Characteristics of the Study Sample and Rates of Diagnostic Errors

Our sample consisted of 302 837 emergency hospitalizations for target conditions (145 729 males [48.1%]; 113 737 participants aged 65 to 74 years [37.6%]; 29 949 Black participants [9.9%]; 246 796 White participants [81.5%]). Additional sample sizes and beneficiary characteristics by condition are presented in the Table. Of the emergency hospitalizations, 13 550 (4.5%) had a potential diagnostic error in the previous 9 days, ranging from 686 potential diagnostic errors per 20 385 hospitalizations for spontaneous intracranial hemorrhage (3.4%) to 67 potential diagnostic errors per 395 emergency hospitalizations for spinal abscess (17.0%) (eTable 1 in Supplement 1). These values were similar when accounting for hospital random effects (eTable 2 in Supplement 1).

**Table.** Characteristics of Medicare Beneficiaries Ages 65 Years and Older With Hospitalization for Selected High-Risk, Emergency Conditions

Characteristic	Emergency admissions, No. (%) (n = 302 837)
Condition, No.	
Ischemic stroke	122 289 (40.4)
Acute myocardial infarction	114 123 (37.7)
Pulmonary embolism	32 988 (10.9)
Spontaneous intracranial hemorrhage	20 385 (6.7)
Arterial thrombosis	3272 (1.1)
Aortic aneurysm	3150 (1.0)
Subarachnoid hemorrhage	2757 (0.9)
Aortic dissection	1843 (0.6)
Meningitis or encephalitis	1635 (0.5)
Spinal abscess	395 (0.1)
Age category, y	
65-74	113 737 (37.6)
75-84	110 745 (36.6)
≥85	78 355 (25.9)
Sex	
Male	145 729 (48.1)
Female	157 108 (51.8)
Race or ethnicity	
Asian or Pacific Islander	6271 (2.1)
Black	29 949 (9.9)
Hispanic	13 976 (4.6)
North American Native	1330 (0.4)
Other^a^	2107 (0.7)
Unknown	2408 (0.8)
White	246 796 (81.5)
Medicaid eligibility	
Not eligible	244 723 (80.8)
Eligible	58 114 (19.2)
HCC risk score, mean (SD)	1.35 (1.2)
Chronic condition prevalence	
Alzheimer disease and related dementias	56 642 (18.7)
Congestive heart failure	82 401 (27.2)
Depression	59 599 (19.7)
Diabetes	118 914 (39.3)
Ischemic heart disease	144 595 (47.8)

Abbreviation: HCC, hierarchical condition category.

^a^ Other race or ethnicity is defined as the other code value in the Master Beneficiary Summary File beneficiary race code modified using the Research Triangle Institute algorithm.

### Estimating Adjusted Rates of Potential Diagnostic Errors

**Figure 1.**
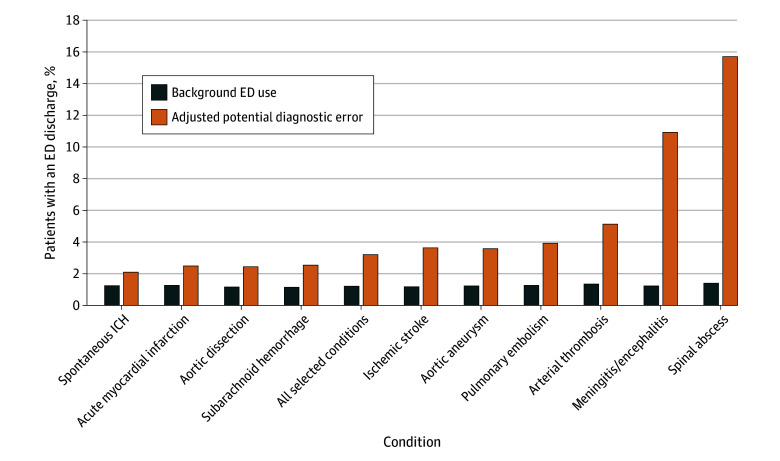
Adjusted Rates of Potential Diagnostic Error for Selected High-Risk Conditions and Background Emergency Department (ED) Utilization for a Comparison Cohort, Using a 9-Day Look-Back Period For each emergency admission, potential diagnostic error, the percentage that were seen in the ED and discharged in the preceding 9 days, was calculated. The background ED utilization rate was defined by the percentage of beneficiaries in a random 20% sample with an ED discharge in the 9 days preceding a randomly selected date. The adjusted rate of potential diagnostic error in the preceding 9 days is the difference between the potential diagnostic error and background ED utilization rate. ICH indicates intracranial hemorrhage.

In the comparison cohort, HCC risk scores ranged from 0.80 to 1.60, with background ED visit rates generally higher for beneficiaries with higher HCC scores (eFigure 1 in Supplement 1). For comparison beneficiaries with an HCC risk score from 1.30 to less than 1.35 (corresponding to the mean value for our emergency hospitalizations cohort), 1.2% (95% CI, 1.2%-1.3%) had an ED discharge in the 9 days preceding a random date. Subtracting this background 9-day ED utilization rate from the 4.5% potential diagnostic error rate for the emergency hospitalizations cohort, we estimate an adjusted diagnostic error rate of 3.2% (95% CI, 3.1%-3.3%). In other words, our best estimate of the population incidence of diagnostic error for Medicare beneficiaries admitted for the selected diagnoses is 3.2%. However, there was variation by condition. Eight conditions had rates at or under 5% (Figure 1 and eTable 1 in Supplement 1), with the lowest for spontaneous intracranial hemorrhage (2.1%; 95% CI, 1.9% to 2.4%). The highest rates were for meningitis (10.9%; 95% CI, 9.3% to 12.5%) and spinal abscess (15.6%; 95% CI, 11.9% to 19.3%). eFigure 2 in Supplement 1 shows the potential diagnostic error, background and adjusted rates out to 90 days.

### Association Between Potential Diagnostic Errors and Mortality

**Figure 2.**
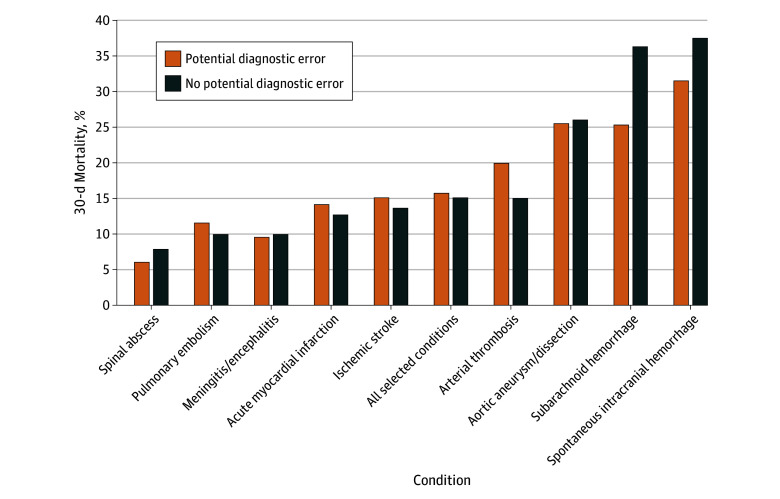
Association Between a Potential Diagnostic Error and Adjusted 30-Day Mortality Overall and by Condition Linear probability models with 30-day mortality as the outcome and presence of a prior emergency department visit (ie, potential diagnostic error) as the binary estimator. Models incorporated hospital random effects, principal admission diagnosis, and year, as well as patient age, sex, Medicaid eligibility, and 25 individual chronic conditions.

Beneficiaries with a potential diagnostic error had higher adjusted 30-day mortality compared with those without a potential diagnostic error (15.7% vs 14.9%; point absolute adjusted difference, 0.8 percentage points; 95% CI, 0.2 to 1.4 percentage points; *P* = .007) (Figure 2). However, the association varied by individual condition. For acute myocardial infarction, stroke, arterial thrombosis, and PE, having a potential diagnostic error was associated with higher 30-day mortality (Figure 2 and eTable 3 in Supplement 1). Potential diagnostic errors were associated with lower 30-day mortality for spontaneous intracranial hemorrhage (31.5% vs 37.5%, difference, −6.0 percentage points; 95% CI, −9.6 to −2.4 percentage points; *P* = .001) and subarachnoid hemorrhage (25.3% vs 36.3%; difference, −11.0 percentage points; 95% CI, −20.1 to −1.8 percentage points; *P* = .02). For the remaining 3 conditions, there was no significant association with mortality.

### Association Between Potential Diagnostic Errors and HDAH

**Figure 3.**
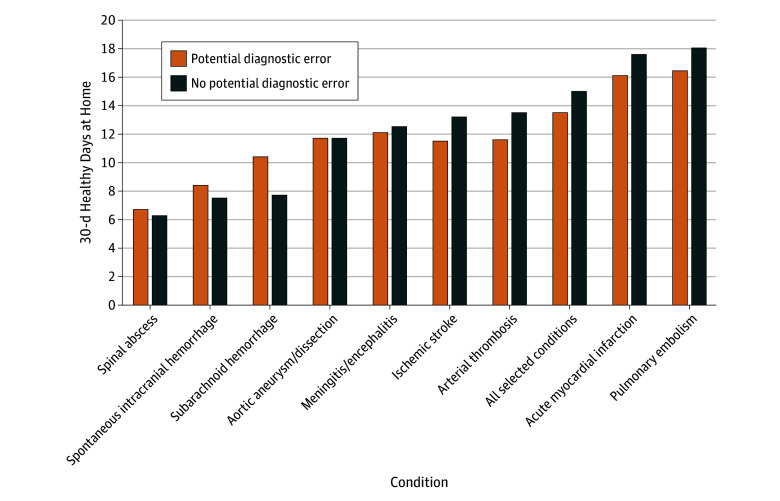
Association Between a Potential Diagnostic Error and Adjusted 30-Day Healthy Days at Home Overall and by Condition Linear regression models with 30-day healthy days at home as the outcome and presence of a prior emergency department visit (ie, potential diagnostic error) as the binary estimator. Models incorporated hospital random effects, principal admission diagnosis, and year, as well as patient age, sex, Medicaid eligibility, and 25 individual chronic conditions.

We similarly found that having a potential diagnostic error was associated with fewer HDAH at 30 days (13.5 vs 15.0 days; difference, −1.4 days; 95% CI, −1.6 to −1.3 days; *P* < .001) for selected conditions in aggregate. When stratified, this association remained significant for acute myocardial infarction, ischemic stroke, PE and arterial thrombosis (Figure 3 and eTable 4 in Supplement 1). For subarachnoid hemorrhage, potential diagnostic error was associated with a greater number of HDAH at 30 days compared with patients who were admitted on the initial ED visit (10.4 vs 7.7 days; difference, 2.8 days; 95% CI, 0.7 to 4.8 days; *P* = .007). A similar association was observed for spontaneous intracranial hemorrhage. There were no significant associations for the remaining conditions.

### Sensitivity Analyses

When we examined intervals of 14 and 30 days, we found adjusted rates of potential diagnostic errors of 4.0% and 5.3%, respectively, for selected conditions in aggregate, again with between-condition variation (eTables 5 and 6 in Supplement 1). For example, for acute stroke admissions, 11 884 of 122 289 strokes (9.7%) had a potential diagnostic error from which 4.0% (95% CI, 3.9%-4.1%) background ED utilization was subtracted to yield an adjusted diagnostic error rate of 5.7% (95% CI, 5.5%-5.9%). Our findings were robust to alternative specifications of hospital transfers (eTable 7 in Supplement 1). When incorporating hospital fixed effects, potential diagnostic error was associated with higher 30-day mortality (15.6% vs 14.8%; point difference, 0.7 percentage points; 95% CI, 0.2 to 1.4 percentage points; *P* = .01) and fewer HDAH (13.4 vs 14.9 days; difference, −1.5 day; 95% CI, −1.6 to −1.3 days; *P* < .001).

## Discussion

In this national study of Medicare fee-for-service beneficiaries with emergent hospitalizations for 10 high-risk conditions, the adjusted rate of potential diagnostic error in the prior 9 days accounting for background ED utilization was 3.2%, although there was substantial variation by condition. Eight conditions had adjusted rates at or under 5%, but higher rates were observed for meningitis and spinal abscess. High-risk emergency hospitalizations with a potential diagnostic error had higher 30-day mortality and fewer HDAH in aggregate, although the associations varied among individual conditions.

We observed relatively low adjusted rates of potential diagnostic error for most high-risk conditions, despite the diagnostic challenges facing emergency physicians in chaotic EDs and despite examining an older population with higher incidence of serious conditions (and potentially atypical presentations). Trying to achieve perfect diagnostic accuracy is not realistic, and it can lead to harm.^15^ For each encounter, physicians must weigh the risks of overtesting (eg, pain, anxiety, allergic reactions, downstream testing associated with incidental findings)^25,26,27^ with the potential benefit of diagnostic information. Although reducing low-value testing and improving diagnostic yield has been encouraged to improve health care safety and value,^28,29^ optimal diagnostic yield remains undefined. Prior studies of acute myocardial infarction^30,31^ and PE^32^ defined an acceptable rate of diagnostic error as less than 2%, a threshold that was felt to represent the ideal balance between the harms of overtesting and the risk of missed diagnosis. While the adjusted estimate of potential diagnostic error for PE in the present study was 4.0%, several conditions had rates ranging from 2% to 3%, suggesting limited room for improvement.

However, meningitis and spinal abscess had substantially higher rates of potential diagnostic error and understanding how to further reduce misdiagnosis for these conditions is warranted. Solutions must be thoughtfully implemented to avoid unintended consequences. Efforts to reduce misdiagnosis in spinal abscess have led to increased testing without improved diagnostic accuracy.^33^ This finding is consistent with a larger body of work suggesting that more testing does not necessarily lead to better diagnostic accuracy or outcomes.^34,35^ Judicious resource use is particularly important for conditions requiring tests that are costly, time-consuming or not readily available across all US EDs, such as magnetic resonance imaging. The diagnosis of meningitis requires an invasive procedure (lumbar puncture). A recent study^36^ of 21 community EDs in Northern California found a decline in lumbar puncture and an increase in computed tomography angiography use for ED patients with headache, with no corresponding change over time in diagnostic error rates for meningitis or subarachnoid hemorrhage. Potential approaches to reducing misdiagnosis include understanding the practice patterns of high-performing clinicians and hospitals, applying machine-learning to augment physician decision-making,^37,38^ and focusing on systems issues (eg, crowding, safety culture) that contribute.^34^

Our study builds on prior work by providing contemporary US-based estimates across a broad range of high-risk conditions using a large national sample. Our estimates of potential diagnostic errors are generally lower than what was estimated in the AHRQ report, but similar to earlier research on cardiovascular conditions.^39^ The present study used the look-back approach similar to the Symptom-Disease Pair Analysis of Diagnostic Error method, which may harness the advantages of large administrative datasets to study diagnostic accuracy for specific diseases.^8^ However, our approach included all prior ED discharges without limiting to specific diagnoses (given that diseases can have atypical presentations)^40,41,42^ and instead adjusted for background ED utilization. A 2009 multistate study found 12.7% of stroke admissions had prior ED discharges with a diagnosis other than cerebrovascular disease.^24^ In our study, 9.7% of stroke admissions had an ED discharge within the prior 30 days, but after accounting for background ED utilization the adjusted 30-day diagnostic error rate was 5.7%. A Michigan study^9^ of PE found similar prior ED visit rates (4.9% vs our 5.3%), with the authors concluding that this likely overestimates diagnostic error as the disease was likely absent on some of the initial visits. Adjusting for background ED utilization, we estimate 4.0% of PE hospitalizations had a potential diagnostic error within the prior 9 days. However, it’s unclear how many were preventable. Return visits may flag potential diagnostic errors, but chart review remains essential to confirm and assess preventability.^14^

The current study builds on prior work by further examining the association between potential diagnostic error and clinical outcomes. While we found that potential diagnostic error was associated with higher 30-day mortality and fewer HDAH for most emergency admissions, the association was only significant for 4 conditions—and was inverse for spontaneous intracranial hemorrhage and subarachnoid hemorrhage. In other words, admissions for these hemorrhages preceded by a potential diagnostic error had lower mortality and more HDAH compared with patients admitted on initial presentation. These findings are likely due to lower severity patients being at greater risk of misdiagnosis and lower risk of death compared with high severity patients, who may be more obviously ill at initial presentation. This phenomenon has been demonstrated in studies of subarachnoid hemorrhage, in which misdiagnosed patients had lower mortality and less severe initial presentations.^43,44^ However, in analyses restricted to patients with lowest initial hemorrhage severity, misdiagnosed patients had worse outcomes.^44,45^

### Limitations

This study has limitations. First, we cannot definitively conclude that ED visits preceding the emergency admissions were true diagnostic errors because administrative claims lack clinical details. However, we use an HCC-stratified cohort to account for background ED utilization and estimate disease-specific diagnostic error rates without needing to determine visit relatedness. As we focused on a subset of particularly high-risk conditions, it is likely that the overall risk of major diagnostic error is smaller for a broader set of conditions. The study includes only Medicare beneficiaries 65 years or older, limiting generalizability to younger populations. However, Medicare beneficiaries account for nearly 25% of ED visits in the US.^46^ Additionally, the advantages of this dataset include the ability to identify adverse outcomes that occur outside of hospital setting; many national datasets including younger individuals cannot reliably identify mortality occurring outside of a health care encounter. Patients who were discharged from the ED and died before returning to the hospital will not be captured by these data but are likely a small proportion of cases. Additionally, if lower acuity patients were more likely to be misdiagnosed on the initial visit, then the association between potential diagnostic errors and higher 30-day mortality may be an underestimate. Also, these data predate the COVID-19 pandemic, which may have increased diagnostic error rates due to health care system strain.

## Conclusions

In this cohort study of Medicare beneficiaries with emergent hospitalizations for selected high-risk emergency conditions, approximately 3.2% were potentially missed on an ED visit within the prior 9 days, with significant variation by condition. Potential diagnostic errors were associated with higher 30-day mortality and fewer HDAH on average, highlighting the importance of efforts to improve diagnostic accuracy in emergency care.

## Notes

Supplement 1.
eAppendix.
**eFigure 1. **Background 9-Day Emergency Department (ED) Utilization, Stratified by Beneficiary Hierarchical Condition Category (HCC) Score **eFigure 2. **Rates of Potential Diagnostic Error, Background Emergency Department (ED) Utilization and Adjusted Potential Diagnostic Error by Day **eTable 1. **Rates of Potential Diagnostic Error, Background Emergency Department (ED) Utilization and Adjusted Potential Diagnostic Error for Medicare Beneficiaries Ages ≥65 Years With Hospitalizations for Selected High-Risk, Emergency Conditions Using a 9-Day Look-Back Period **eTable 2. **Rates of Potential Diagnostic Error Accounting for Hospital Random Effects **eTable 3. **Association Between a Potential Diagnostic Error and Adjusted 30-Day Mortality by Condition **eTable 4. **Association Between a Potential Diagnostic Error and Adjusted 30-day Healthy Days at Home (HDAH) by Condition **eTable 5. **Rates of Potential Diagnostic Error, Background Emergency Department (ED) Utilization and Adjusted Potential Diagnostic Error for Medicare Beneficiaries Ages ≥65 Years With Hospitalizations for Selected High-Risk, Emergency Conditions Using a 14-Day Look-Back Period **eTable 6. **Rates of Potential Diagnostic Error, Background Emergency Department (ED) Utilization and Adjusted Potential Diagnostic Error for Medicare Beneficiaries Ages ≥65 Years With Hospitalizations for Selected High-Risk, Emergency Conditions Using a 30-Day Look-Back Period **eTable 7. **Association Between Potential Diagnostic Error and 30-Day Mortality and 30-Day Healthy Days at Home (HDAH) Using Alternative Definition of Interhospital Transfer

Supplement 2.
Data Sharing Statement
