# Supplementary material for: Potential Diagnostic Error for Emergency Conditions, Mortality, and Healthy Days at Home
Source: JAMA Netw Open. 2025 Jun 17;8(6):e2516400. doi: 10.1001/jamanetworkopen.2025.16400 (PMC12175027; doi:10.1001/jamanetworkopen.2025.16400)
Supplement: Supplement 1. — eAppendix. eFigure 1. Background 9-Day Emergency Department (ED) Utilization, Stratified by Beneficiary Hierarchical Condition Category (HCC) Score eFigure 2. Rates of Potential Diagnostic Error, Background Emergency Department (ED) Utilization and Adjusted Potential Diagnostic Error by Day eTable 1. Rates of Potential Diagnostic Error, Background Emergency Department (ED) Utilization and Adjusted Potential Diagnostic Error for Medicare Beneficiaries Ages ≥65 Years With Hospitalizations for Selected High-Risk, Emergency Conditions Using a 9-Day Look-Back Period eTable 2. Rates of Potential Diagnostic Error Accounting for Hospital Random Effects eTable 3. Association Between a Potential Diagnostic Error and Adjusted 30-Day Mortality by Condition eTable 4. Association Between a Potential Diagnostic Error and Adjusted 30-day Healthy Days at Home (HDAH) by Condition eTable 5. Rates of Potential Diagnostic Error, Background Emergency Department (ED) Utilization and Adjusted Potential Diagnostic Error for Medicare Beneficiaries Ages ≥65 Years With Hospitalizations for Selected High-Risk, Emergency Conditions Using a 14-Day Look-Back Period eTable 6. Rates of Potential Diagnostic Error, Background Emergency Department (ED) Utilization and Adjusted Potential Diagnostic Error for Medicare Beneficiaries Ages ≥65 Years With Hospitalizations for Selected High-Risk, Emergency Conditions Using a 30-Day Look-Back Period eTable 7. Association Between Potential Diagnostic Error and 30-Day Mortality and 30-Day Healthy Days at Home (HDAH) Using Alternative Definition of Interhospital Transfer [file jamanetwopen-e2516400-s001.pdf]

Supplemental Online Content

Lin MP, Burke RC, Sabbatini AK, et al. Potential diagnostic error for emergency conditions and association with mortality and healthy days at home. *JAMA Netw Open*. 2025;8(6):e2516400
doi:10.1001/jamanetworkopen.2025.16400

eAppendix.

eFigure 1. Background 9-Day Emergency Department (ED) Utilization, Stratified by Beneficiary Hierarchical Condition Category (HCC) Score

eFigure 2. Rates of Potential Diagnostic Error, Background Emergency Department (ED) Utilization and Adjusted Potential Diagnostic Error by Day

eTable 1. Rates of Potential Diagnostic Error, Background Emergency Department (ED) Utilization and Adjusted Potential Diagnostic Error for Medicare Beneficiaries Ages ≥65 Years With Hospitalizations for Selected High-Risk, Emergency Conditions Using a 9-Day Look-Back Period

eTable 2. Rates of Potential Diagnostic Error Accounting for Hospital Random Effects

eTable 3. Association Between a Potential Diagnostic Error and Adjusted 30-day Mortality by Condition

eTable 4. Association Between a Potential Diagnostic Error and Adjusted 30-day Healthy Days at Home (HDAH) by Condition

eTable 5. Rates of Potential Diagnostic Error, Background Emergency Department (ED) Utilization and Adjusted Potential Diagnostic Error for Medicare Beneficiaries Ages ≥65 Years With Hospitalizations for Selected High-Risk, Emergency Conditions Using a 14-Day Look-Back Period

eTable 6. Rates of Potential Diagnostic Error, Background Emergency Department (ED) Utilization and Adjusted Potential Diagnostic Error for Medicare Beneficiaries Ages ≥65 Years With Hospitalizations for Selected High-Risk, Emergency Conditions Using a 30-Day Look-Back Period

eTable 7. Association Between Potential Diagnostic Error and 30-Day Mortality and 30-Day Healthy Days at Home (HDAH) Using Alternative Definition of Interhospital Transfer

This supplemental material has been provided by the authors to give readers additional information about their work.

© 2025 Lin MP et al. *JAMA Network Open*.

eAppendix.

A) Diagnosis Codes for Selected High-Risk Conditions: We identified the following conditions, that were featured in the AHRQ report on diagnostic error as being top conditions associated with serious misdiagnosis-related harm. Index emergency hospitalizations were identified in Medicare claims using ICD10 codes as shown below. We excluded conditions in the AHRQ report that we felt were clinically not necessarily requiring a diagnosis in the ED (e.g., lung cancer, cardiac arrythmia) and/or lacking clearly defined ICD10 diagnosis codes (sepsis, intestinal obstruction, intestinal perforation).

**Diagnosis**	**Description**	**ICD10**
Ischemic stroke	Cerebral infarction	I63
	Stroke, not specified as hemorrhage or infarction	I64
Spontaneous intracranial hemorrhage	Other nontraumatic intracranial hemorrhage	I62
	Intracerebral hemorrhage	I61
Subarachnoid hemorrhage		I60
Acute myocardial infarction		I21
Aortic dissection		171.0
Aortic aneurysm		I71.1 171.2 I71.3 I71.4 I71.5 I71.6 171.8 I71.9
Arterial thrombosis	Arterial embolism and thrombosis	I74
Pulmonary embolism	Pulmonary embolism with acute cor pulmonale	I26.0
	Pulmonary embolism without acute cor pulmonale	I26.9
Meningitis/encephalitis	Meningitis	G00 G01 G02 G03 A87 A0221 A170 A2781 A390 A5041 A5141
	Encephalitis	G04 G05 A83 A84 A85 A86
Spinal abscess	Intraspinal abscess and granuloma	G06.1

The following conditions that were included in the AHRQ report but not the present study are as follows.

© 2025 Lin MP et al. *JAMA Network Open*.

1) **Lung cancer-** this condition was excluded because clinically it is not necessarily a diagnosis that must be established in the ED but may be deferred to the ambulatory setting.

2) **Pneumonia-** It is common for viral infections to evolve to pneumonia and thus the presence of an ED visit prior to a pneumonia admission is likely to represent evolution of the disease process rather than diagnostic error in many cases, compared to other diseases included in the report. Additionally, pneumonia does not necessarily require admission and is often much a lower acuity condition compared to other conditions in the study (e.g., aortic disease, spinal abscess).

3) **Sepsis-** Sepsis is a challenging to study using claims data, with well-documented limitations of claims data to accurately study this disease including upcoding and lack of data on sepsis severity.

4) **Spinal cord compression-** this diagnosis was excluded given lack of clearly defined ICD10 codes for emergent cord compression requiring ED diagnosis and surgical management.

5) **Intestinal obstruction-** there is a heterogeneous set of causes of intestinal obstruction. Accordingly, there are numerous associated ICD 10 codes and the less severe paralytic ileus may also be included in these codes.

B) Identification of prior ED visits as indicators of potential diagnostic errors
Prior research has proposed an approach to identifying ED visits that uses both facility and professional claims,[1] as there are circumstances in which an ED visit may generate either a professional or facility claim but not both. Particularly relevant to this study is the Centers for Medicare and Medicaid Services rule that related outpatient diagnostic and therapeutic services occurring within 3 days of an inpatient stay be bundled with the inpatient claim. This policy raises the concern that some prior ED visits may not be identified using facility claims alone and may lead to an underestimation of diagnostic error. We thus employed a technique using both facility and professional claims, the latter of which are not affected by this payment policy. Our approach was as follows:

1) **We first identified ED discharges prior to admission using facility claims.** Consistent with prior work,[2,3] we identified outpatient claims with ED revenue center codes (0450-0459, 0981). We removed claims ending observation (revenue center codes of 0760, 0761, 0762, and 0769).

2) **Next, we identified professional claims for ED visits that were not identified by facility claims.** This was a multi-stage process, as outlined below.

    a. We identified all professional claims with a place of service code of emergency room (23) and HCPCS/CPT codes for ED evaluation and management (99281-99285) or critical care 99291 prior to admission and up to 3 days after.
    b. Consistent with prior research,[1] claims with identical tax identification number, performing provider (NPI number), and dates of service were considered duplicates.
    c. After inspection of the data, we identified other circumstances that we deemed to be duplicate claims and not separate visits:
        i. If there were duplicate claims with the same date of service but one claim had a payment amount of $0, the non-zero payment claim was kept. Claims with the same date of service but one of the claims had a payment amount of $0. The non-zero payment claim was kept.
        ii. Claims with the same date of service but one of the clinician specialties is not typically associated with working in the ED (e.g. neurology vs. the typical specialties of emergency medicine, nurse practitioner and physician assistant), claim with the typical provider was kept.
        iii. Claims with the same date of service, but one of the claims had a modifier of "GC" (service was performed by a resident), them the non-resident claim was kept.

    d. We identified inpatient and outpatient claims with ED revenue center codes (0450-0459, 0981) prior to admission.

© 2025 Lin MP et al. *JAMA Network Open*.

e. We then linked the ED professional and facility claims (including the index admission) by date of service. A professional claim that overlapped within 3 days of a facility claim was matched and considered a single ED visit. If there were multiple matches, we performed matching without replacement; the professional services claim with the closest start date to a facility claim start date was matched. All remaining unmatched professional claims were considered unique ED visits not captured by the facility claims and were added to the original facility-only data produced in step 1.

3) Estimated the number of prior ED discharges preceding an inpatient stay by day.

a. We considered visits on the same or preceding calendar days as likely transfers or related to the index inpatient stay in our primary analysis.
b. We assigned an ED disposition of discharge to outpatient facility claims that did not end in observation or death, consistent with prior work.[2,3] ED professional claims without match to a facility claim were assumed to be ED discharges.

4) Compared ED visits by day preceding an emergency inpatient stay using a facility only vs. facility + professional claims approach.

a. We plotted the number of emergency admissions that had a prior ED visit within the preceding 2 to 90 days and compared the estimates using a facility only vs. facility + professional claim approach to identifying ED visits.
b. As shown in the Figure below, a facility only approach appears to underestimate ED discharges in the three days preceding an admission, likely due to the Medicare policy of bundling outpatient services within 3 days of an inpatient stay with the inpatient stay. For this reason, we used the facility + professional claims approach to estimate diagnostic error as represented by preceding ED visits.

Emergency discharges preceding an index admission by day and identification method

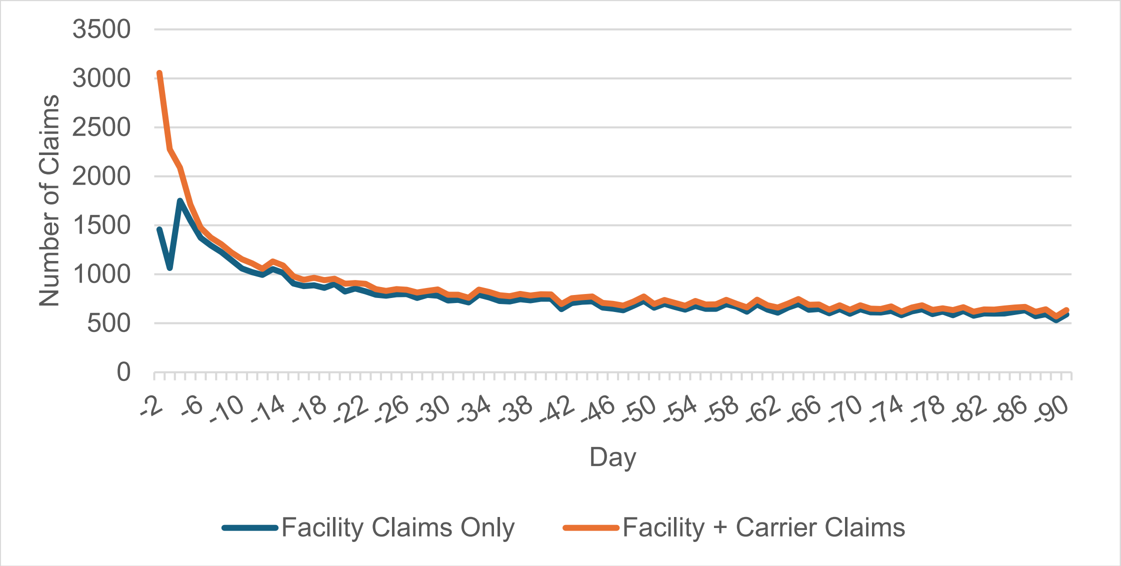

C. Identification of transfers. The primary analysis excluded all ED visits on the same and prior day as likely transfers or duplicate records, given the known limitations of transfer status in claims data.[4] Additionally, the primary analysis includes events that generate only a professional claim for ED services, which do not have a disposition variable at all. Thus, we performed a sensitivity analysis in which we identified prior ED within 9 days using only facility claims and comparing the two definitions of transfer (excluding same or prior day visits vs. excluding only those prior outpatient ED visits with a discharge destination of "transfer" (PTNT_DSCHRG_STUS_CD= 02 or 05).

© 2025 Lin MP et al. *JAMA Network Open*.

eFigure 1. Background 9-day Emergency Department (ED) Utilization, Stratified by Beneficiary Hierarchical Condition Category (HCC) Score

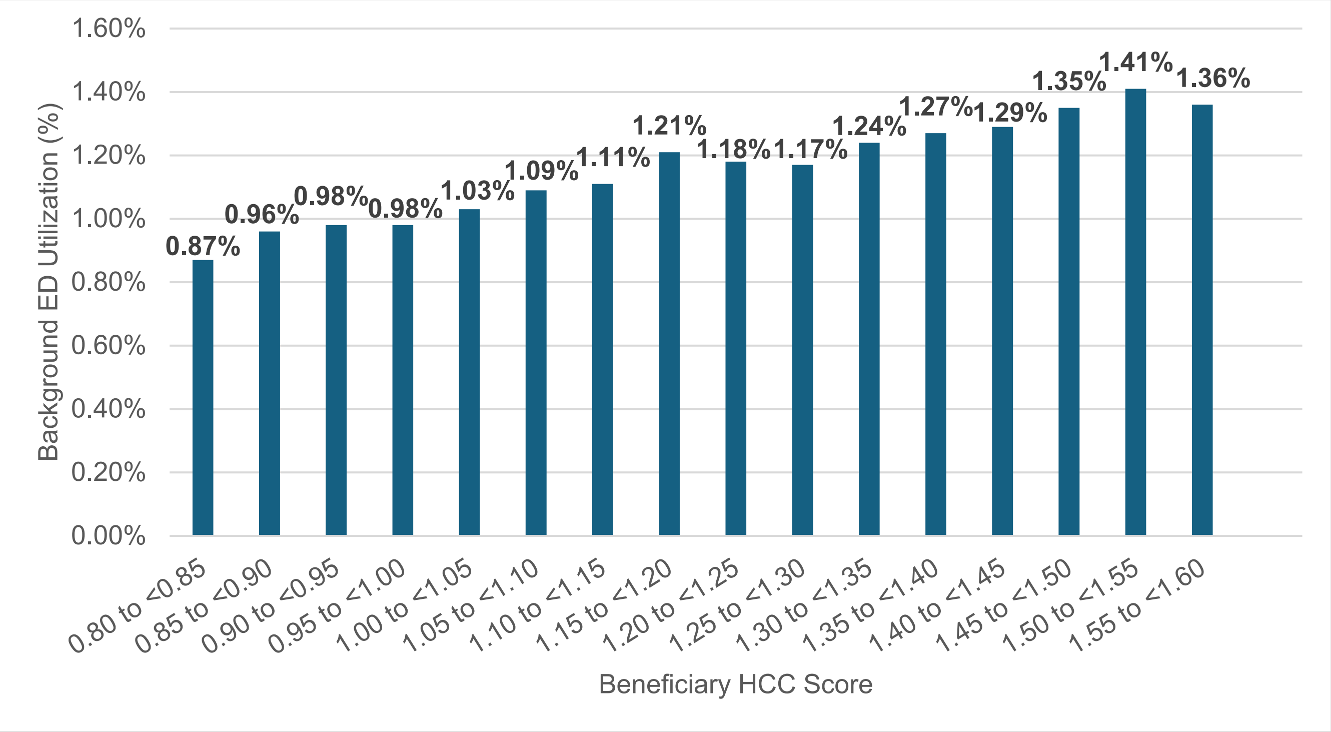

We identified beneficiary Centers for Medicare and Medicaid Services Hierarchical Condition Category (HCC) scores, which are associated with healthcare utilization and mortality. Both CCW conditions and HCC scores were identified from the prior calendar year to avoid adjusting for changes in health status that occurred during or after the index hospitalization. Background ED utilization is defined by the percentage of randomly selected dates among a comparison cohort of beneficiaries with at least one ED discharge in the preceding 9 days. This 9-day ED discharge rate is shown by bin of beneficiary HCC score.

© 2025 Lin MP et al. *JAMA Network Open*.

eFigure 2. Rates of Potential Diagnostic Error, Background Emergency Department (ED) Utilization and Adjusted Potential Diagnostic Error by Day

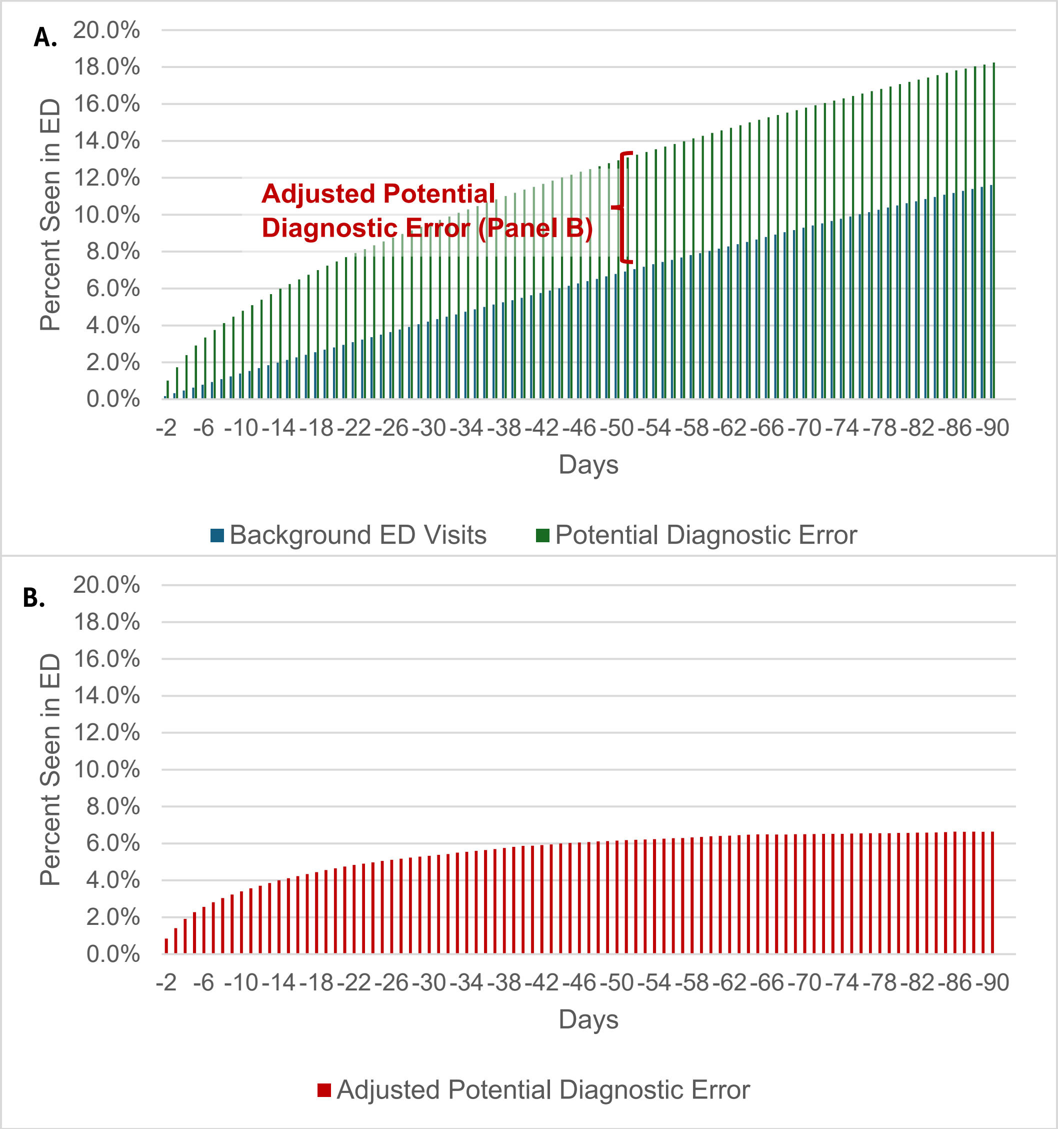

Panel A shows the daily cumulative rates of potential diagnostic error (percentage of emergency admissions with a preceding ED discharge) as well as background ED utilization (percentage of randomly selected dates among a comparison cohort of beneficiaries with a preceding ED discharge). The difference between the two is the adjusted potential diagnostic error rate (shown in panels A and B).

© 2025 Lin MP et al. *JAMA Network Open*.

eTable 1. Rates of Potential Diagnostic Error, Background Emergency Department (ED) Utilization and Adjusted Potential Diagnostic Error for Medicare Beneficiaries Ages ≥65 Years With Hospitalizations for Selected High-Risk, Emergency Conditions Using a 9-Day Look-Back Period

	Mean HCC score	A<br>Potential Diagnostic Error (%)	B<br>Background ED Utilization (%)	C<br>Adjusted Potential Diagnostic Error (%)
All Selected Conditions	1.35	4.47%	1.24%	3.23%
Acute Myocardial Infarction	1.40	3.78%	1.27%	2.51%
Aortic Aneurysm	1.27	4.86%	1.17%	3.69%
Aortic Dissection	1.21	3.74%	1.18%	2.56%
Arterial Thrombosis	1.57	6.39%	1.36%	5.03%
Ischemic Stroke	1.29	4.91%	1.17%	3.74%
Meningitis/Encephalitis	1.37	12.17%	1.27%	10.90%
Pulmonary Embolism	1.36	5.29%	1.27%	4.02%
Spinal Abscess	1.52	16.96%	1.41%	15.55%
Spontaneous Intracranial Hemorrhage (ICH)	1.37	3.37%	1.27%	2.10%
Non-Traumatic Subarachnoid Hemorrhage (SAH)	1.14	3.81%	1.11%	2.70%

Adjusted diagnostic error rates (C) were calculated by subtracting the background prior ED visit rate (B) for the corresponding HCC risk group from the potential diagnostic error rates (column A, i.e., observed prior ED visit rate in the emergency admissions sample). We did this for emergency admissions overall as well as individually for each condition. Prior ED discharges with the same diagnosis as the index admission were not considered a diagnostic error.

© 2025 Lin MP et al. *JAMA Network Open*.

eTable 2. Rates of Potential Diagnostic Error Accounting for Hospital Random Effects

	9 Days with 95% CI (%)	14 Days with 95% CI (%)	30 Days with 95% CI (%)
All Selected Conditions	4.57 (4.48, 4.66)	6.14 (6.03, 6.25)	9.83 (9.68-9.97)
Acute Myocardial Infarction	3.86 (3.73 3.99)	5.18 (5.03, 5.34)	8.47 (8.28, 8.67)
Aortic Aneurysm	4.95 (4.23, 5.68)	6.76 (5.92, 7.59)	9.93 (8.90, 10.96)
Aortic Dissection	3.86 (2.92, 4.81)	5.17 (4.09, 6.26)	8.55 (7.21, 9.90)
Arterial Thrombosis	6.50 (5.79, 7.21)	9.20 (8.38, 10.01)	14.52 (13.51, 15.53)
Ischemic Stroke	5.01 (4.88, 5.13)	6.53 (6.38, 6.68)	10.00 (9.81, 10.19)
Meningitis/Encephalitis	12.26 (11.26, 13.26)	15.16 (14.01, 16.31)	20.48 (19.06, 21.90)
Pulmonary Embolism	5.38 (5.15, 5.61)	7.76 (7.50, 8.03)	13.08 (12.75, 13.41)
Spinal Abscess	17.02 (14.98, 19.06)	22.14 (19.80, 24.48)	32.39 (29.50, 35.28)
Spontaneous Intracranial Hemorrhage (ICH)	3.48 (3.19, 3.77)	5.05 (4.71, 5.38)	9.16 (8.74, 9.58)
Non-Traumatic Subarachnoid Hemorrhage (SAH)	3.92 (3.15, 4.70)	5.00 (4.11-5.89)	7.83 (6.73, 8.93)

Linear probability models with potential diagnostic error (i.e., a prior emergency department discharge for a different diagnosis) as the outcome and incorporating hospital random effects.

© 2025 Lin MP et al. *JAMA Network Open*.

eTable 3. Association Between a Potential Diagnostic Error and Adjusted 30-day Mortality by Condition

	Admissions with a Potential Diagnostic Error	Admissions without a Potential Diagnostic Error	Adjusted Difference in 30-Day Mortality	P-Value
All Selected Conditions	15.7%	14.9%	0.8% (0.22%, 1.42%)	0.007
Acute Myocardial Infarction	14.1%	12.7%	1.5% (0.45%, 2.46%)	0.004
Aortic Aneurysm/Dissection	25.5%	26.0%	-0.4% (-6.20%, 5.31%)	0.88
Arterial Thrombosis	19.9%	14.9%	5.0% (0.04%, 10.03%)	0.048
Ischemic Stroke	14.9%	13.6%	1.3% (0.40%, 2.15%)	0.004
Meningitis/Encephalitis	9.5%	10.0%	-0.4% (-4.77%, 3.91%)	0.85
Pulmonary Embolism	11.5%	9.9%	1.6% (0.15%, 3.03%)	0.03
Spinal Abscess	6.0%	7.8%	-1.8% (-9.17%, 5.49%)	0.62
Spontaneous Intracranial Hemorrhage (ICH)	31.5%	37.5%	-6.0% (-9.65%, -2.39%)	0.001
Non-Traumatic Subarachnoid Hemorrhage (SAH)	25.3%	36.3%	-11.0% (-20.14%, -1.80%)	0.02

*Linear probability models with 30-day mortality as the outcome and the presence of a potential diagnostic error (i.e., a preceding ED discharge for a different diagnosis) as the binary predictor. Models incorporated hospital random effects, principal admission diagnosis, and year, as well as patient age, sex, Medicaid eligibility and 25 individual chronic conditions.

© 2025 Lin MP et al. *JAMA Network Open*.

eTable 4. Association Between a Potential Diagnostic Error and Adjusted 30-day Healthy Days at Home (HDAH) by Condition

	Admissions with a Potential Diagnostic Error	Admissions without a Potential Diagnostic Error	Adjusted Difference in 30-Day Mortality (95% CI)	P-Value
All Selected Conditions	13.5	15.0	-1.4 (-1.62, -1.26)	<0.001
Acute Myocardial Infarction	16.1	17.6	-1.5 (-1.81, -1.19)	<0.001
Aortic Aneurysm/Dissection	11.72	11.70	0.02 (-1.49, 1.54)	0.98
Arterial Thrombosis	11.6	13.5	-1.8 (-3.38, -0.31)	0.02
Ischemic Stroke	11.5	13.2	-1.7 (-2.03, -1.46)	<0.001
Meningitis/Encephalitis	12.10	12.52	-0.4 (-1.93, 1.09)	0.58
Pulmonary Embolism	16.5	18.1	-1.6 (-2.09, -1.11)	<0.001
Spinal Abscess	6.7	6.3	0.4 (-2.09, 2.80)	0.77
Spontaneous Intracranial Hemorrhage (ICH)	8.4	7.5	0.9 (0.11, 1.66)	0.02
Non-Traumatic Subarachnoid Hemorrhage (SAH)	10.4	7.7	2.8 (0.74, 4.76)	0.007

*Linear regression models with 30-day HDAH as the outcome and presence of a potential diagnostic error (i.e., a preceding ED discharge for a different diagnosis) as the binary predictor. Models incorporated hospital random effects, principal admission diagnosis, and year, as well as patient age, sex, Medicaid eligibility and 25 individual chronic conditions.

© 2025 Lin MP et al. *JAMA Network Open*.

eTable 5. Rates of Potential Diagnostic Error, Background Emergency Department (ED) Utilization and Adjusted Potential Diagnostic Error for Medicare Beneficiaries Ages ≥65 Years With Hospitalizations for Selected High-Risk, Emergency Conditions Using a 14-Day Look-Back Period

	Mean HCC score	A Potential Diagnostic Error (%)	B Background ED Utilization (%)	C Adjusted Potential Diagnostic Error (%)
All Selected Conditions	1.35	5.99%	1.99%	4.00%
Acute Myocardial Infarction	1.40	5.05%	2.05%	3.00%
Aortic Aneurysm	1.27	6.60%	1.89%	4.71%
Aortic Dissection	1.21	4.99%	1.88%	3.11%
Arterial Thrombosis	1.57	9.02%	2.19%	6.83%
Ischemic Stroke	1.29	6.38%	1.89%	4.49%
Meningitis/Encephalitis	1.37	15.05%	2.05%	13.00%
Pulmonary Embolism	1.36	7.62%	2.05%	5.57%
Spinal Abscess	1.52	22.03%	2.16%	19.87%
Spontaneous Intracranial Hemorrhage (ICH)	1.37	4.87%	2.05%	2.82%
Non-Traumatic Subarachnoid Hemorrhage (SAH)	1.14	4.82%	1.80%	3.02%

Adjusted diagnostic error rates (C) were calculated by subtracting the background prior ED visit rate (B) of the corresponding HCC risk group from the potential diagnostic error rates (column A, i.e., observed prior ED visit rate in the emergency admissions sample). We did this for emergency admissions overall as well as individually for each condition. Prior ED discharges with the same diagnosis as the index admission were not considered a potential diagnostic error.

© 2025 Lin MP et al. *JAMA Network Open*.

eTable 6. Rates of Potential Diagnostic Error, Background Emergency Department (ED) Utilization and Adjusted Potential Diagnostic Error for Medicare Beneficiaries Ages ≥65 Years With Hospitalizations for Selected High-Risk, Emergency Conditions Using a 30-Day Look-Back Period

	Mean HCC score	A<br>Potential Diagnostic Error (%)	B<br>Background ED Utilization (%)	C<br>Adjusted Potential Diagnostic Error (%)
All Selected Conditions	1.35	9.54%	4.20%	5.34%
Acute Myocardial Infarction	1.40	8.23%	4.40%	3.83%
Aortic Aneurysm	1.27	9.65%	3.99%	5.66%
Aortic Dissection	1.21	8.19%	3.98%	4.21%
Arterial Thrombosis	1.57	14.18%	4.58%	9.60%
Ischemic Stroke	1.29	9.72%	3.99%	5.73%
Meningitis/Encephalitis	1.37	20.24%	4.40%	15.84%
Pulmonary Embolism	1.36	12.82%	4.40%	8.42%
Spinal/Intracranial Abscess	1.52	32.15%	4.63%	27.52%
Spontaneous Intracranial Hemorrhage (ICH)	1.37	8.82%	4.40%	4.42%
Non-Traumatic Subarachnoid Hemorrhage (SAH)	1.14	7.51%	3.92%	3.59%

Adjusted diagnostic error rates (C) were calculated by subtracting the background prior ED visit rate (B) of the corresponding HCC risk group from the potential diagnostic error rates (column A, i.e., observed prior ED visit rate in the emergency admissions sample). We did this for emergency admissions overall as well as individually for each condition. Prior ED discharges with the same diagnosis as the index admission were not considered a potential diagnostic error.

© 2025 Lin MP et al. *JAMA Network Open*.

eTable 7. Association Between Potential Diagnostic Error and 30-Day Mortality and 30-Day Healthy Days at Home (HDAH) Using Alternative Definition of Interhospital Transfer

	Association with 30-Day Mortality[a]			Association with 30-day HDAH[b]		
	**Potential Diagnostic Error**	**No Potential Diagnostic Error**	**Difference (95% CI)**	**Potential Diagnostic Error**	**No Potential Diagnostic Error**	**Difference (95% CI)**
Transfers defined as those ED visits on the same or preceding days as the index admission[c] (prior ED visit rate=3.4%)	16.2%	14.9%	1.3% (0.59%, 1.96%)	13.6 days	14.9 days	-1.4 (-1.61, -1.19)
Transfers defined using the discharge status code [d] (prior ED visit rate= 4.5%)	16.1%	14.9%	1.2% (0.60%, 1.79%)	13.7 days	15.0 days	-1.2 (-1.41, -1.05)

[a] Linear probability models with 30-day mortality as the outcome and presence of a potential diagnostic error (i.e., a preceding ED discharge for a different diagnosis) as the binary predictor. [b] Linear regression models with 30-day HDAH as the outcome and a potential diagnostic error as the binary predictor. All models incorporated hospital random effects, principal admission diagnosis, and year, as well as patient age, sex, Medicaid eligibility and 25 individual chronic conditions. Identification of prior ED visits as indicators of potential diagnostic error was limited to facility claims, as professional claims do not have a variable for visit disposition/discharge destination. ED discharges were identified in facility claims only and any outpatient ED visits within 1 calendar day of the index admission were considered transfers and not diagnostic errors. [d] In this alternative definition of transfer, the patient discharge status code of transfer on the outpatient facility claim was used to identify transfers to another hospital.

© 2025 Lin MP et al. *JAMA Network Open*.

References

1. Venkatesh AK, Mei H, Kocher KE, et al. Identification of Emergency Department Visits in Medicare Administrative Claims: Approaches and Implications. *Acad Emerg Med*. Apr 2017;24(4):422-431. doi:10.1111/acem.13140
2. Burke LG, Epstein SK, Burke RC, Orav EJ, Jha AK. Trends in Mortality for Medicare Beneficiaries Treated in the Emergency Department From 2009 to 2016. *JAMA Intern Med*. 01 01 2020;180(1):80-88. doi:10.1001/jamainternmed.2019.4866
3. Burke LG, Burke RC, Epstein SK, Orav EJ, Jha AK. Trends in Costs of Care for Medicare Beneficiaries Treated in the Emergency Department From 2011 to 2016. *JAMA Netw Open*. 08 03 2020;3(8):e208229. doi:10.1001/jamanetworkopen.2020.8229
4. Nikpay S, Leeberg M, Kozhimannil K, et al. A proposed method for identifying Interfacility transfers in Medicare claims data. *Health Serv Res*. Feb 2025;60(1):e14367. doi:10.1111/1475-6773.14367

© 2025 Lin MP et al. *JAMA Network Open*.
