# Supplementary material for: Potential Diagnostic Error for Emergency Conditions, Mortality, and Healthy Days at Home
Source: JAMA Netw Open. 2025 Jun 17;8(6):e2516400. doi: 10.1001/jamanetworkopen.2025.16400 (PMC12175027; doi:10.1001/jamanetworkopen.2025.16400)
Supplement: Supplement 2. — Data Sharing Statement [file jamanetwopen-e2516400-s002.pdf]

Data Sharing Statement

Lin. Potential Diagnostic Error for Emergency Conditions, Mortality, and Healthy Days at Home. *JAMA Netw Open*. Published June 17, 2025.
doi:10.1001/jamanetworkopen.2025.16400

Data
Data available: No

Additional Information
Explanation for why data not available: These data are CMS data and data use agreements prohibit sharing. Code will be made available.
